# A study on giant panda recognition based on images of a large proportion of captive pandas

**DOI:** 10.1002/ece3.6152

**Published:** 2020-03-10

**Authors:** Peng Chen, Pranjal Swarup, Wojciech Michal Matkowski, Adams Wai Kin Kong, Su Han, Zhihe Zhang, Hou Rong

**Affiliations:** ^1^ Chengdu Research Base of Giant Panda Breeding Chengdu China; ^2^ Sichuan Key Laboratory of Conservation Biology for Endangered Wildlife Chengdu China; ^3^ Sichuan Academy of Giant Panda Chengdu China; ^4^ School of Computer Science and Engineering Nanyang Technological University Singapore City Singapore; ^5^ College of Computer Science Sichuan Normal University Chengdu China

**Keywords:** giant panda, individual identification, panda face recognition, population estimation

## Abstract

1. As a highly endangered species, the giant panda (panda) has attracted significant attention in the past decades. Considerable efforts have been put on panda conservation and reproduction, offering the promising outcome of maintaining the population size of pandas. To evaluate the effectiveness of conservation and management strategies, recognizing individual pandas is critical. However, it remains a challenging task because the existing methods, such as traditional tracking method, discrimination method based on footprint identification, and molecular biology method, are invasive, inaccurate, expensive, or challenging to perform. The advances of imaging technologies have led to the wide applications of digital images and videos in panda conservation and management, which makes it possible for individual panda recognition in a noninvasive manner by using image‐based panda face recognition method.
2. In recent years, deep learning has achieved great success in the field of computer vision and pattern recognition. For panda face recognition, a fully automatic deep learning algorithm which consists of a sequence of deep neural networks (DNNs) used for panda face detection, segmentation, alignment, and identity prediction is developed in this study. To develop and evaluate the algorithm, the largest panda image dataset containing 6,441 images from 218 different pandas, which is 39.78% of captive pandas in the world, is established.
3. The algorithm achieved 96.27% accuracy in panda recognition and 100% accuracy in detection.
4. This study shows that panda faces can be used for panda recognition. It enables the use of the cameras installed in their habitat for monitoring their population and behavior. This noninvasive approach is much more cost‐effective than the approaches used in the previous panda surveys.

## Graphical abstract

This study shows that panda faces can be used for panda recognition. It enables the use of the cameras installed in their habitat for monitoring their population and behaviour. This noninvasive approach that leverages on deep learning is much more cost‐effective than the approaches used in the previous panda surveys.

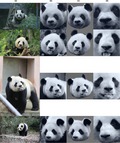

## 1 INTRODUCTION

Population size is an important factor determining whether species can persist in nature and also an important indicator of regional biodiversity (McNeely, Miller, Reid, Mittermeier, & Werner, 1990). Accurate estimation of their population sizes is crucial for developing effective conservation and management schemes. (Miller, Joyce, & Waits, 2005; Smallwood & Schonewald, 1998; Solberg, Bellemain, Drageset, Taberlet, & Swenson, 2006; Zhan et al., 2006). Ecologists have been trying to identify individual animals, including giant panda (*Ailuropoda melanoleuca*) to accurately estimate their population and to study their spatial behavior. (Xiangjiang et al., 2009) This information is vital for developing suitable animal protection strategies (Pollard, Blumstein, & Griffin, 2010; Zheng et al., 2016). The giant panda population and its dynamics are not only the basis for delineating nature reserves, establishing local conservation management institutions, and establishing ecological corridor zones but also the important indicators for evaluating the effectiveness of conservation management schemes. Also, they reflect the vulnerability of ecosystems in the area of study. In practice, it is hard to accurately estimate the population size of giant pandas because of their small and sparsely distributed population in large habitats with complex forests and mountains. It is difficult to find their tracks.

For effectively protecting giant pandas, since 1974, four panda surveys have been conducted by the National Forestry and Grassland Administration, China to estimate their population size and distribution and collect other related information. According to the fourth population survey, Sichuan Province with the largest panda population has 1,387 wild pandas with a density of 0.0684 individuals per km square. Nearly 700 field scientists from more than 100 organizations participated in the fourth survey. The traditional survey methods, including direct counting method, route survey method, and distance‐bite discrimination method, and the molecular biological methods based on the feces of pandas, such as DNA fingerprint detection technology and microsatellite analysis, were employed. (State Forestry Administration, 2006, 2015a; Zhan et al., 2006). Although the molecular biological methods can accurately separate different pandas, its effectiveness is strictly limited by the freshness of samples (Zhan et al., 2006). Both traditional survey methods and molecular biological methods are difficult to carry out on a large scale in a short period, because they require enormous human, material, and financial resources, and the success rate of sample acquisition is low. Therefore, more cost‐effective and accurate panda population survey methods are still in demand.

Fortunately, the advances in imaging equipment, computer vision, and machine learning technologies, including deep neural networks^1^ (LeCun, Bengio, & Hinton, 2015) make it possible to effectively and efficiently analyze animals based on images. Cameras have been installed in the habitats of giant pandas (Kelly, 2008), and therefore, computer vision and machine learning technologies can be applied to analyze and monitor their behavior and population.

### 1.1 Image‐based animal recognition

Recent studies have deployed deep neural networks to analyze animals in images for conservation, wildlife biology and zoology applications. Willi et al. used a convolutional neural network (CNN) to identify different species in images collected from camera traps (Willi et al., 2019). The authors combined CNN and the citizen science approach, in which registered volunteers manually annotate images online, to reduce the manual effort and time of ecology researchers. Norouzzadeh et al. investigated the potential of CNN to automatically identify, count, and describe animals in a camera trap image (Norouzzadeh et al., 2018). Contrary to the works, which study automatically distinguishing between different species (Norouzzadeh et al., 2018; Willi et al., 2019), in this work, the terms, recognition, and identification, refer to distinguishing between individual animals in the same species.

Related studies showed that image‐based methods have great potential in wild animal recognition. A recent review of different methods for animal recognition can be found in (Schneider, Taylor, Linquist, & Kremer, 2019). Some animals like zebra, tiger, and giraffe have highly unique patterns composed of stripes, patches, or spots on their coats, which benefit the image‐based animal recognition (Burghardt & Campbell, 2007; Cheema & Anand, 2017; Kumar & Singh, 2016). However, pandas have a very similar appearance in terms of coat patterns, which makes it challenging to distinguish them based on images. To address this problem, researchers proposed to utilize panda faces for recognition (Matkowski et al., 2019).

Animal recognition based on facial biometrics has been studied to meet various demands of different applications. The recognition of livestock, such as pigs and cows, is an important part of precision agriculture. Hansen et al. applied three face recognition methods on a pig dataset and reported promising results (Hansen et al., 2018). Animal face recognition was also proposed for wild animal tracking. Freytag et al. proposed a face recognition algorithm built on CNN for wild chimpanzee recognition (Freytag et al., 2016). Deb et al. (2018) proposed a specially designed identification algorithm based on CNN for several endangered primates, including golden monkeys, lemurs, and chimpanzees. Schofield et al. studied chimpanzee face detection, tracking, and recognition from videos taken in an outdoor chimpanzee field site (Schofield et al., 2019).

Some image‐based studies have been conducted on pandas. Zhang, Sun, and Tang (2010) proposed a head detection method employing gradient oriented features for several animals, including cat, tiger, and panda. Chen et al. proposed an example‐based approach and a topology modelling based method for panda facial region detection (Chen, Wen, Qu, & Mete, 2012; Chen, Wen, Zhuo, & Mete, 2012a). Chen et al. developed a method based on gradient shapes for estimating the pose of pandas in images (Chen, Wen, Zhuo, & Mete, 2012b). Our preliminary work is the first attempt to use facial biometrics for automatic panda recognition (Matkowski et al., 2019), but it has several limitations. The database used in the study had only 28 pandas and 163 images. It is significantly smaller than the database established for this study. Furthermore, it has a strict input image requirement. The images should only contain panda face.

### 1.2 Scope of this study

The scope of the study is panda identification on a much larger dataset and a more automatic and realistic setting. A new dataset consisting of 6,441 fontal panda face images from 218 different pandas is established. The dataset includes 39.78% of captive pandas in the world. It is well‐known that the number of pandas all over the world is very limited. According to the fourth (last) panda survey report published by the State Forestry Administration, Beijing, China, in 2015 (State Forestry Administration, 2015b), there were in total 1864 pandas in the wild. Additionally, almost no control was imposed on imaging environments and the pandas in the data acquisition. These are fundamental challenges in panda recognition and the differences from human face recognition, where extremely large face image databases are available, and control over data acquisition and user cooperation can be expected in some applications.

Inspired by the success of deep neural networks in the computer vision field, a panda face recognition algorithm based on deep neural networks is developed. The proposed algorithm performs a sequence of operations, including panda face detection, segmentation, alignment, and identification. In other words, the algorithm is fully automatic. It is evaluated in two settings, closed‐set identification, where all testing pandas have images in the training set and open‐set identification, where some testing pandas have no images in the training set. In the closed‐set identification setting, the algorithm only returns a list of panda identities and the corresponding probabilities, which indicate how likely the panda in a testing image is a particular panda in the training set. In the open‐set identification setting, in addition to the list of panda identities and the corresponding probabilities, the algorithm also needs to decide whether the panda in an input image is one of the pandas in the training set or not, because the panda may be an unseen panda.

The improvements of the proposed algorithm and this study over the preliminary work in Matkowski et al. (2019) are as follows. (a) In this study, the input images are raw images with pandas and diverse backgrounds whereas in the preliminary work, the input images are panda faces only. (b) The proposed algorithm is fully automatic; no manual segmentation or detection is needed. (c) The preliminary work is based on traditional hand‐crafted features, while the proposed algorithm relies on state‐of‐the‐art deep networks. (d) The proposed algorithm is evaluated on the largest panda face image dataset collected in different scenarios in closed‐set and open‐set identification settings. According to the best knowledge of the authors, it is the only study investigating panda face identification in this scale and the fully automatic setting.

## 2 MATERIALS AND METHODS

### 2.1 Dataset

By November 2018, the number of giant pandas in captivity worldwide reached 548 (People's Daily Online). Their detailed individual information, such as genealogy, gender, and breeding records, are managed by the National Forestry and Grassland Administration and the Chinese Association of Zoological Gardens. Thus, it is possible to obtain accurate identity information for this study.

**Figure 1:**
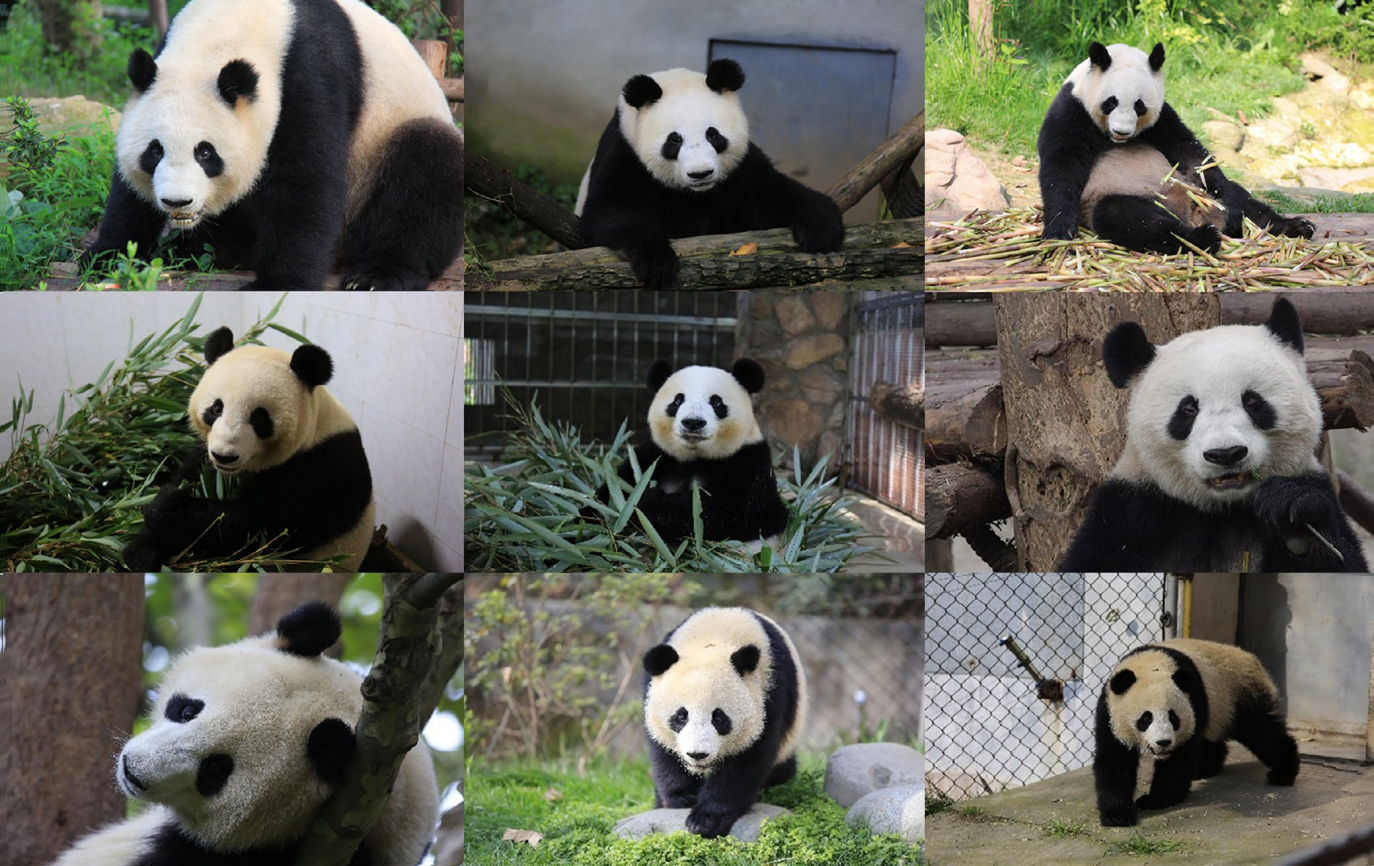
Sample panda images used in this study. Images in each row were collected from the same panda

**Figure 2:**
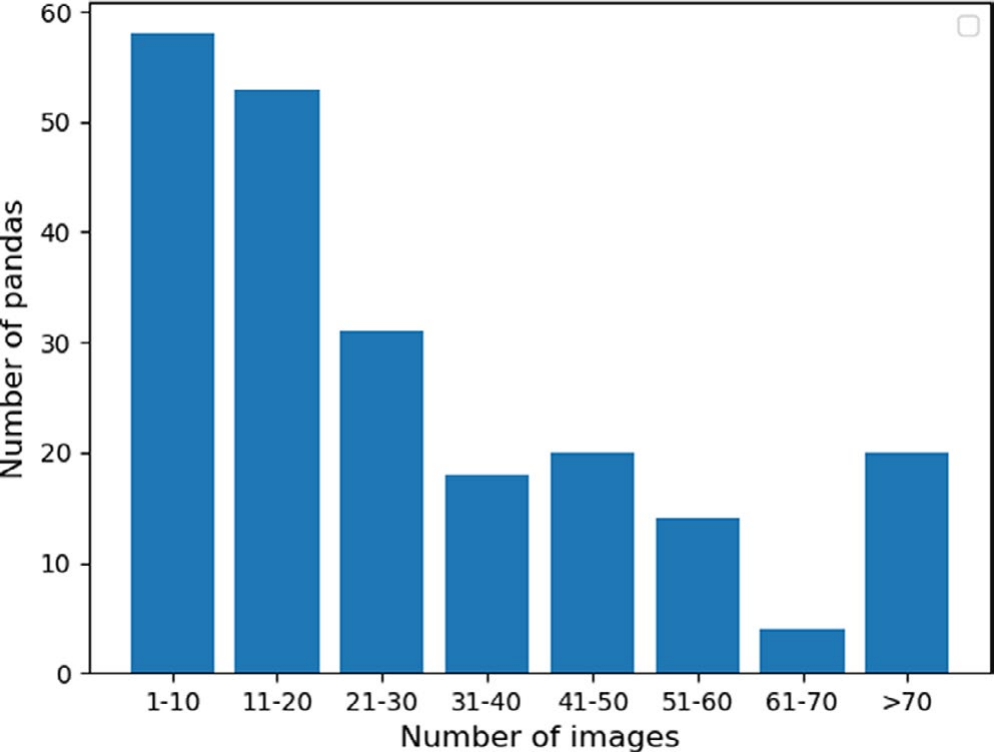
Histogram of the number of images of the 218 pandas

As of 31 December 2018, Chengdu Research Base of Giant Panda Breeding (CRBGPB) has the largest (35.6%) captive giant panda population in the world. For this study, 6,441 images with frontal panda faces from 218 pandas were retrieved from image archives and collected using a Panasonic dvx200 video camera and three cameras—a Canon 1DXmarkII camera, a Canon 5DmarkIII camera, and a Panasonic Lumix DMC‐GH4 camera. These images were taken from a wide range of viewpoints and distance and showed pandas in their routine activities, such as eating bamboos, walking, and lying down. Figure 1 shows sample panda images used in this study, and images in each row were collected from the same panda. In this dataset, different pandas have different numbers of images ranging from 2 images to 168 images. On average, each panda has 29.54 images in the dataset. The histogram in Figure 2 shows the data distribution. The resolution of the images ranges from 8,688 by 5,792 pixels to 440 by 293 pixels. A total of 52.9 percent of the images are in the range between 1,024 by 678 pixels and 1920 by 1,080 pixels.

The images were manually annotated by 15 annotators. The annotation process is divided into two stages. In the first stage, bounding boxes are used to locate panda faces and in the second stage, polygons with on average 44, 14, 12, 14, 12, 10, and 11 vertexes are used to annotate the face, left ear, left eye, right ear, right eye, nose, and mouth, respectively. The bounding boxes and the facial features annotations were used only for training the networks. Manually annotated bounding boxes were used to train the face detection network. Three sets of data were used to train the segmentation and alignment network. Manually cropped images and the segmented image ground truths were, respectively, the input and the target output in training the segmentation network. For the alignment ground truth dataset, images were aligned using an algorithm that used the eyes and mouth annotations to align the cropped and segmented images. Although gender and age information of the pandas are not used in the current algorithm, a summary of the data is given: 3,743 images are collected from female pandas and 2,698 images are collected from male pandas; 328, 4,001, 1,271, and 784 are collected from old (above 20 years old), adult (5.5–20 years old), subadult (1.5–5.5 years old), and juvenile (0–1.5 years old) pandas and 57 images do not have age information.

**Figure 3:**
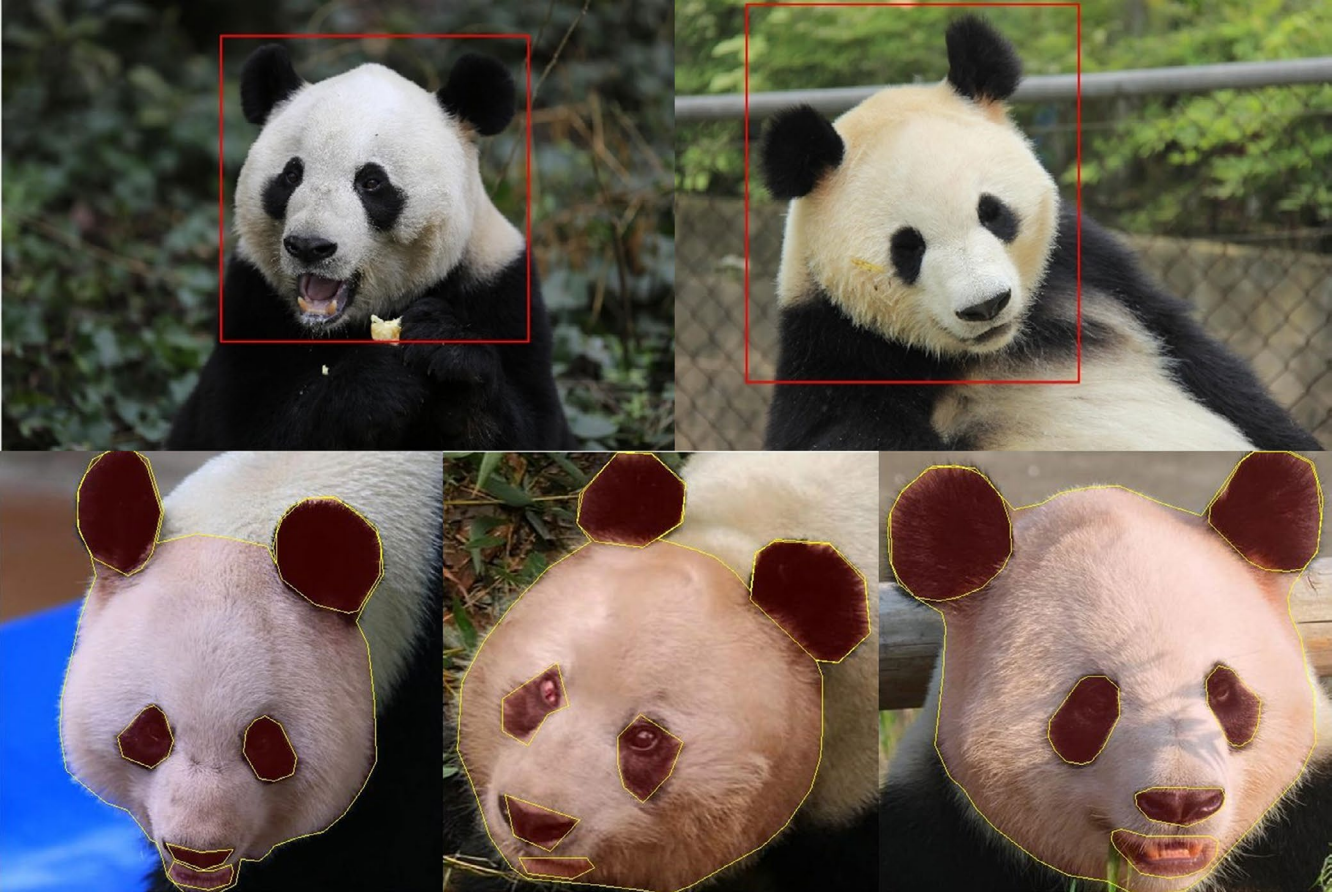
The first and the second rows show, respectively, the results from the first and the second level annotations

During the testing phase, all the tasks, that is, detection, segmentation, alignment, and identification were performed using networks with the original raw images. Figure 1 shows some of these images. Figure 3 shows examples of annotated images.

### 2.2 The proposed panda face recognition algorithm

**Figure 4:**
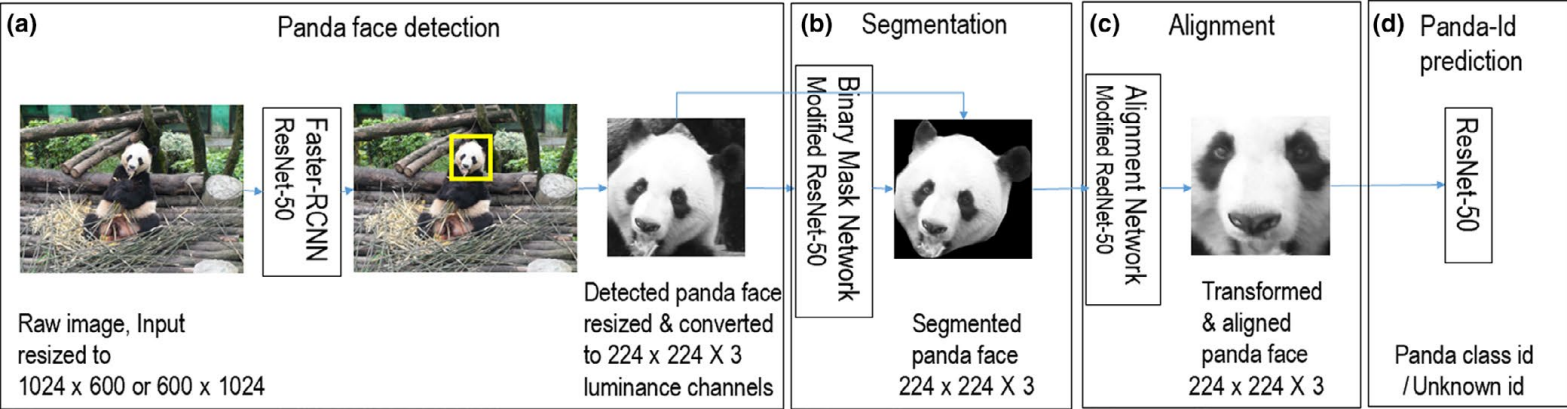
Illustration of the proposed panda face recognition algorithm

Figure 4 illustrates the algorithm. Firstly, a detection network (Girshick, 2015; Liu et al., 2016; Redmon, Divvala, Girshick, & Farhadi, 2016) is applied to raw input images to detect frontal panda faces, which are indicated by output bounding boxes. Secondly, the facial regions within the bounding boxes are extracted and inputted to the image segmentation network (He, Gkioxari, Dollár, & Girshick, 2017; Long, Shelhamer, & Darrell, 2015; Ronneberger, Fischer, & Brox, 2015) and a spatial transformer network (Jaderberg, Simonyan, & Zisserman, & Kavukcuoglu, 2015) for segmentation and alignment, respectively. Finally, normalized face images are fed into a deep network to determine the identity of the panda. In this Section, the model architecture, its components (Section 2.2.1) and training (Section 2.2.2) are briefly discussed. For the more detailed description of the model architecture and training, please refer to the Appendix S1.

#### 2.2.1 Model architecture

##### Detection

**Figure 5:**
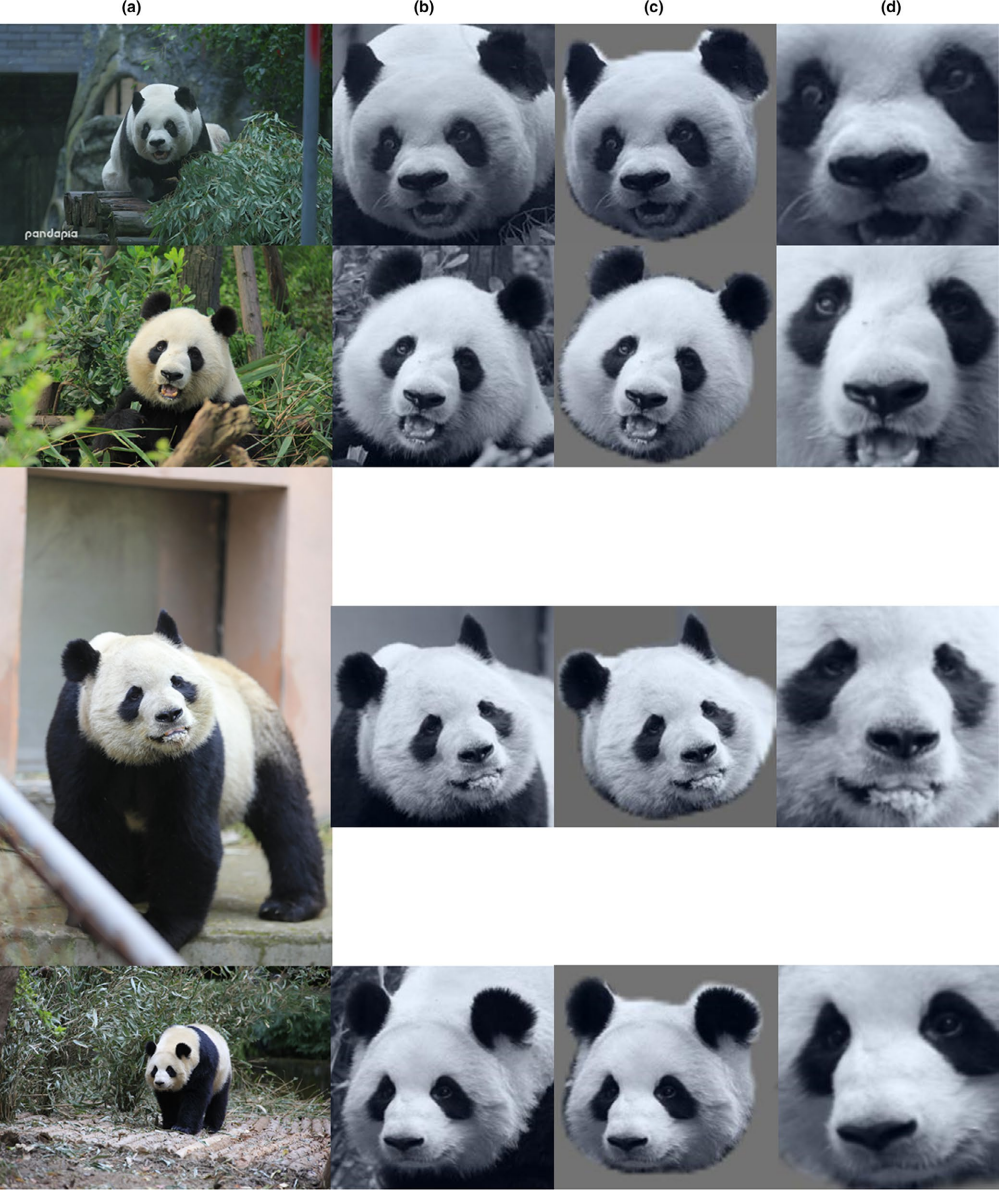
(a) is the raw input images and (b)‐(d) are, respectively, the outputs of the detection, segmentation and alignment networks. The images in (b)–(d) are black and white images

Faster R‐CNN (Ren, He, Girshick, & Sun, 2015) is the state‐of‐the‐art generic object detection algorithm based on deep learning, which consists of a region proposal network and a classification network. The region proposal network returns region candidates which may contain objects. The classification network is used to classify the objects in the region candidates and refine bounding box coordinates to fit the objects more accurately. In the first module, for panda face detection (Figure 4a), Faster R‐CNN, which uses ResNet‐50 layers (He, Zhang, Ren, & Sun, 2016), is employed. This network was already trained using the COCO dataset (Lin et al., 2014) and can be downloaded from the Tensorflow detection model zoo. The pretrained network was fine‐tuned to detect panda faces. The inputs of the Faster R‐CNN are panda images (Figure 5a) which were rescaled to 1,024 by 600 pixels for horizontal images and 600 by 1,024 pixels for vertical images. The Faster R‐CNN outputs bounding box coordinates, which are used to crop panda faces (Figure 5b). The cropped images are detected panda faces, which are resized to 224 by 224 pixels.

##### Segmentation

The second module (Figure 4b) is responsible for background removal by generating a binary mask—an image whose black pixels represent the background and white pixels represent panda face. It uses a ResNet‐50, which was trained on the ImageNet dataset (Deng et al., 2009), and then the top layers after ResNet Layer 3 were pruned. A new convolution layer followed by average pooling, convolution transpose, and activation layers is added on the top. This network takes the cropped panda face image from the Faster R‐CNN and outputs a binary mask, which is element‐wisely multiplied with the input image to produce a segmented panda face image. Figure 5c shows outputs of this network.

##### Alignment

The third module (Figure 4c) determines affine transformation parameters for aligning the segmented panda face image to a reference template in order to minimize rotation, shift and scaling variations among different panda face images. This module contains a ResNet‐50, whose layers are pruned after ResNet Layer 4, followed by average pooling and two fully connected layers with activations. The last layer has six neurons corresponding to six affine transformation parameters, which are used to align the segmented panda face image into a reference panda face. Figure 5c,d show segmented images before and after alignment. The aligned images are passed to the last module for panda ID prediction.

##### Panda identity prediction

The last module (Figure 4d) is a standard classification network. A ResNet‐50, which was trained on the ImageNet dataset and further fine‐tuned on the aligned panda faces, is used to determine the identity of the panda in the input image. Each output node in the last layer gives a probability value indicating how likely the panda in the input image is the panda corresponding to the node. In closed‐set identification, the number of output nodes is equal to the number of pandas in the training set. In this setting, the proposed algorithm does not handle unknown pandas explicitly. If an application environment has unknown pandas, experts need to manually compare the most likely pandas outputted by the algorithm with the panda in the input image. Manual comparison is also used in forensic applications. In open‐set identification, the number of output nodes in the last layer is equal to the number of pandas with known identities with one additional node for pandas with unknown identities. The output of the additional node is a probability value indicating how likely the panda in the input image is not one of the pandas with known identity. More details about the network architectures can be found in the Appendix S1.

#### 2.2.2 Training

All the networks were trained on an Ubuntu 18.04 workstation with Intel Xeon(R) E5‐1650 v4 CPU and NVIDIA GTX 1080 Ti GPU. The code was implemented in Python using Tensorflow and Pytorch. The networks were trained using a supervised approach in two phases. The supervised approach means that each input training sample is associated with a ground truth output, which is used to calculate loss functions for deriving optimal network parameters.

In the first phase, the Faster R‐CNN for panda face detection was trained using pairs of raw images and corresponding ground truth bounding boxes around panda faces. During training, the network took raw images as inputs and outputs predicted bounding boxes, which are used to calculate four different loss functions. After training, this network is used to detect panda faces in images. 5,854 images were used to train the panda face detection network, 185 images were used to validate the model during training and 402 images were used to evaluate the performance of the trained detection network.

In the second phase, all the remaining three modules, that is, the segmentation, alignment, and identification networks, were trained sequentially using the cropped panda face images as inputs. The corresponding ground truths are binary masks, affine transformation parameters, and panda identities, which were used to calculate the corresponding loss functions to derive optimal network parameters. Additionally, data augmentation (spatial and color transformations) was used. It is a common technique to combat model overfitting and enhance network performance on unseen data. To address the imbalanced dataset problem, an augmented dataset was constructed from the images detected using the panda face detection network. New images generated by randomly applying translation, rotation, brightness, contrast, and sharpness operations on a randomly selected image were added in the dataset. These operations were applied on all the pandas, except for the one with maximum number of images. Thus, all pandas in the augmented training dataset had the same number of images. The ground truth bounding boxes, which were manually marked, were used to train the first module, the Faster R‐CNN. Data augmentation was performed on the training dataset. The cropped (from Faster R‐CNN) & resized (224 × 224) images were translated in both horizontal and vertical directions to a maximum of ±15 pixels. The training examples were also rotated in the range of ±20°, randomly. The cropped images after detection were converted to a single‐channel grayscale (224 × 224 × 1), and then the same channel was copied thrice to form (224 × 224 × 3) input for the subsequent network. As the images were converted to grayscale; only sharpness, brightness, and contrast augmentation were applied randomly on the training images. The images were varied to up to ±15%, randomly on the brightness, contrast, and sharpness scales. Finally, after the augmentation, each panda had 134 images. In total, there were 29,212 images in the augmented training dataset. The images in the test dataset and the validation datasets were not augmented. The augmented dataset was used to train the networks in the second phase. After training, these networks work in a sequence to determine panda identities.

The proposed algorithm was trained for closed‐set identification and open‐set identification. For closed‐set identification, the original dataset with 6,441 images was split into a gallery set containing 5,854 images, a validation set containing 185 images, and a probe set containing 402 images. Both gallery and probe sets had images from all the 218 pandas and the images in the validation set were selected from pandas with more than 5 images in the training dataset. Each panda has at least one image in the probe set. Data augmentation mentioned above was applied on the gallery set and created a training set with 29,212 images. For open‐set identification, the gallery set was reorganized. 4,983 images from 176 pandas were augmented to a total of 23,584 images to form a seen panda dataset and 505 images from 20 pandas were augmented to a total of 2,680 images to form an unseen panda dataset for training the networks. In the identification network, the 176 pandas corresponded to 176 output nodes for identifying them, and the 20 pandas corresponded to one output node for detecting unseen panda. Note that 366 images from 22 pandas in the original gallery set were not used in training. 338 images from the 176 pandas, which were considered seen pandas and 29 images from the 22 pandas, which were considered as unseen pandas were used to evaluate the proposed algorithm in the open‐set identification setting. These images were from the original probe set. Details about the training and loss functions can be found in the Appendix S1.

### 2.3 Evaluation metrics

For evaluating image‐based biometric recognition systems, gallery and probe sets are commonly used. The gallery set is a dataset that contains images with known identities. The probe set contains image queries which are input to recognition systems to determine their identities. For each query, the system outputs comparison scores, which indicate similarity/dissimilarity between the query and the identities in the gallery set. All these comparison scores are further used to calculate the performance metrics. Two metrics, receiver operating characteristic (ROC) and cumulative match characteristic (CMC) curves, are commonly used to evaluate recognition system performance.

#### 2.3.1 Receiver operating characteristic

ROC curve shows true positive rates (TPR) against false positive rates (FPR), which measures system accuracy in answering whether a query and a given identity are the same or different individuals (verification). Points on a ROC curve are calculated by thresholding all the comparison scores and calculating the rate of true and false positives for each threshold. It is desirable, for a recognition system to set the threshold at high TPR and low FPR. However, there is a trade‐off between higher TPR and lower FPR, and one must choose a threshold suitable for a target application. To numerically compare two recognition systems based on their ROCs, it is common to report TPRs at few different FPRs, for example, 0.1, 0.01, 0.001, etc., or to report equal error rate (EER), at which 1‐TPR and FPR are equal.

#### 2.3.2 Cumulative match characteristic curve

A CMC curve shows identification rates at different ranks and the point at the *K*th rank indicates the percentage of queries' identities are correctly retrieved within top‐*K* ranks. The CMC curve is calculated by sorting (ascending/descending) comparison scores for each query to determine its rank in the sorted gallery. Usually, rank‐1 (Top‐1) and rank‐5 (Top‐5) identification rates are used to assess and compare the system performance. In some applications, which involve a human to further process the search results, higher ranks, for example, rank‐10 or rank‐30, are also important.

## 3 RESULTS

**Figure 6:**
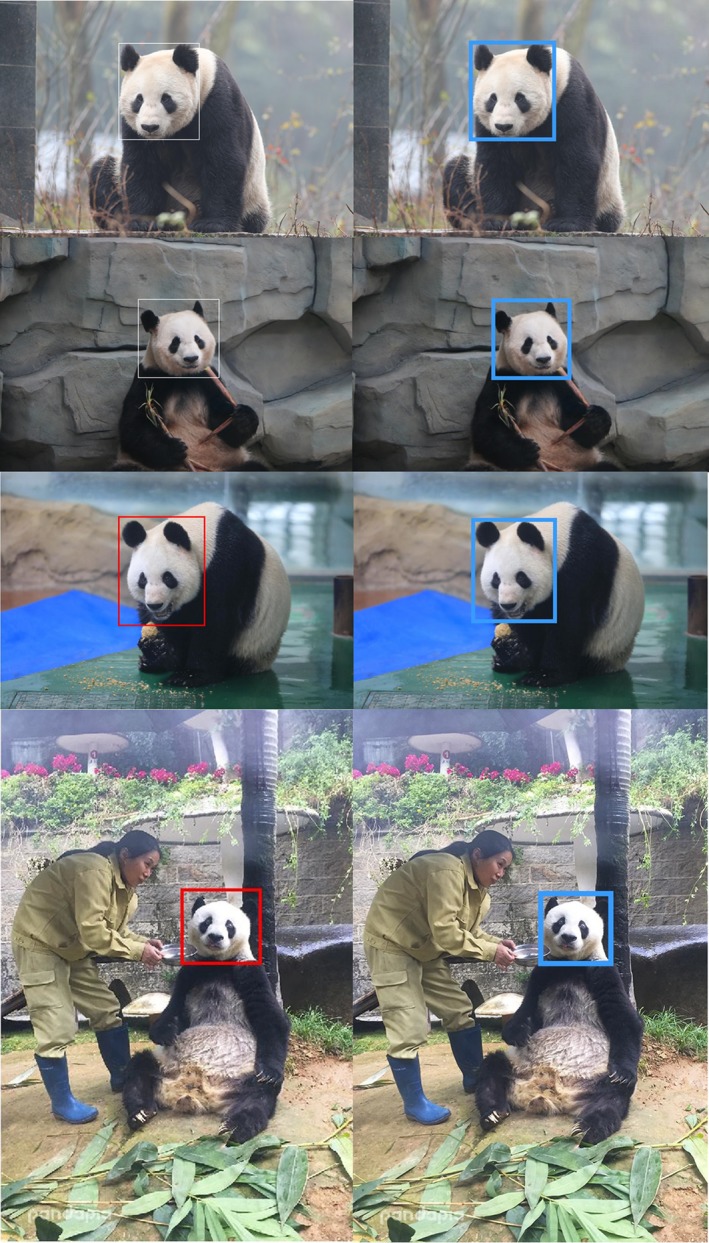
The panda face detection ground truths (left) and results (right)

**Figure 7:**
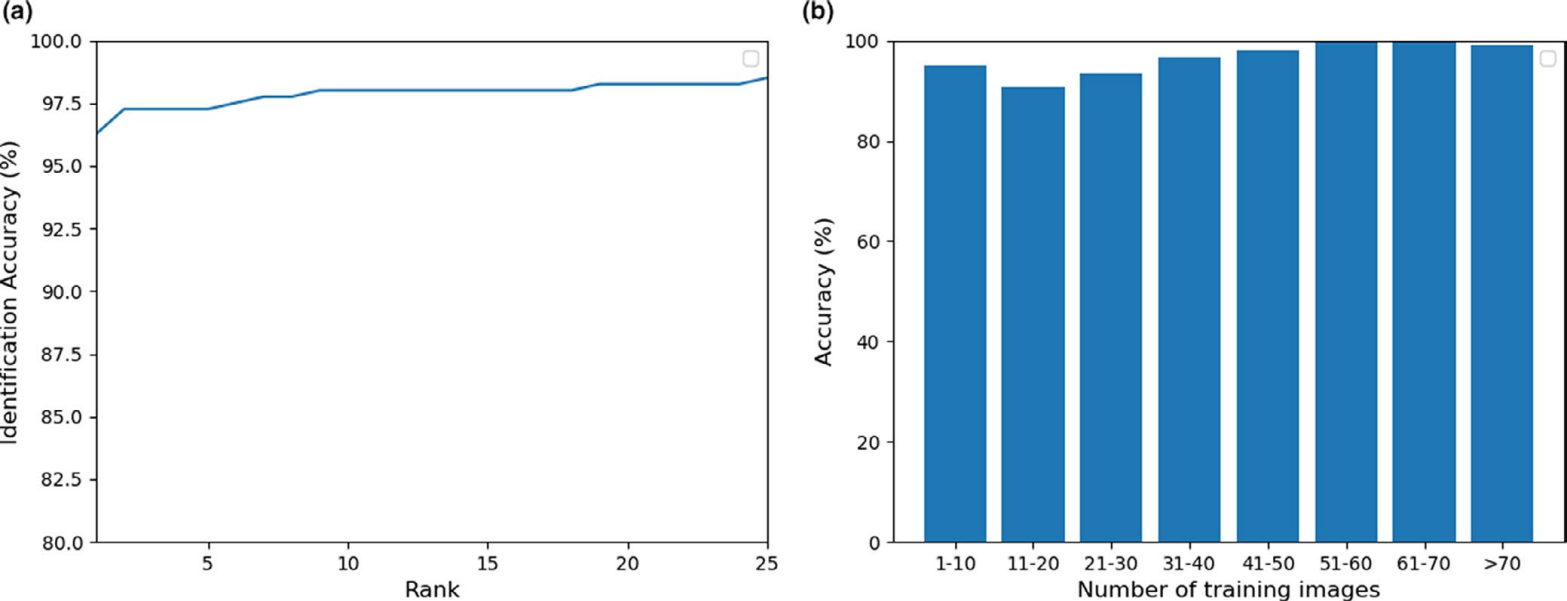
(a) The CMC curve of closed‐set identification and (b) the identification accuracies of pandas with different number of training images

**Figure 8:**
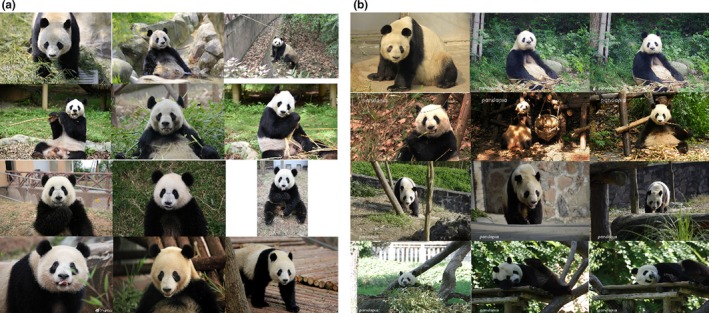
(a) Examples of images where the pandas were correctly identified and (b) examples of images where the pandas were incorrectly identified. Images in each row are from the same panda. The images in the first two columns are from the training set. The images in the last column are from the testing set

Firstly, the detection performance and the closed‐set accuracy are reported. Intersection over Union is an evaluation metric used to measure the detection accuracy of an object detector. A threshold is used to decide if there is a significant intersection between the ground truth bounding box and the predicted bounding box within the combined area covered by the two boxes. If the ratio is above the threshold, then the detected bounding box is assumed to be correct. The detection network achieved 100% accuracy at 70% IoU (intersection over union). Figure 6 shows some detection results. For closed‐set identification, the algorithm achieved a top‐1 accuracy of 96.27% and top‐5 accuracy of 97.25%. Figure 7a shows the resultant CMC curve. Note that Figure 7a shows only 25 ranks, but the gallery set has 218 pandas. Figure 8a,b give, respectively, correctly and incorrectly identified panda images. Figure 8 indicates that image quality has impact on the identification results. The panda faces with occlusion and large pose variations are more challenging. Additional analysis is performed to understand the identification performance. In this analysis, the pandas are grouped based on their numbers of original training images. The identification accuracy in each group is plotted as a bar chart, which is shown in Figure 7b. Figure 7b indicates that more training images likely give high accuracy. It is a common property in machine learning algorithms, in particular, deep learning‐based methods because they are very data‐hungry.

To choose the classification model for the panda identity prediction, an experiment was conducted on Resnet‐101, Restnet‐50, and Resnet‐18 and the results are given in Table 1. Three models were pretrained using the ImageNet dataset. While Resnet‐101 was the deepest model, Resnet‐50 and Resnet‐18 were the shallower versions of the same network architecture. Overall, the results of the Resnet‐50 model with pretrained weights gave the highest Top‐1 accuracy and hence this model was used for all other experiments.

**Table 1:** The closed‐set identification accuracy from three pretrained models

Model	Top‐1 sccuracy (%)
Resnet‐101	95.52
Resnet‐50	96.27
Resnet‐18	95.02

**Figure 9:**
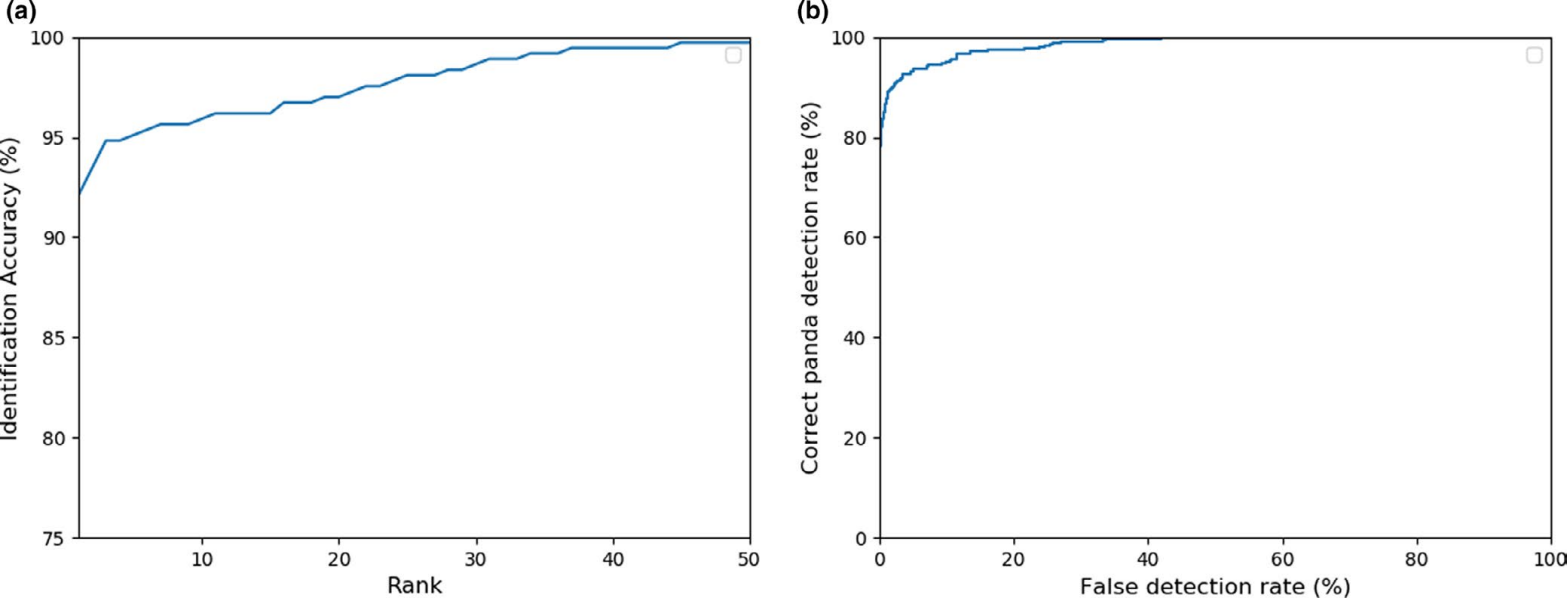
(a) The CMC curve of open‐set identification and (b) the ROC curve of detecting unknown pandas

For open‐set identification, the algorithm achieved a top‐1 accuracy of 92.12% and top‐5 accuracy of 95.09%. Figure 9a shows the resultant CMC curve. The performance of the algorithm drops comparing with closed‐set identification (Figure 7a), because of the reduction of training images and the unknown pandas in the testing set. To see whether the proposed algorithm can detect unknown pandas, different thresholds are applied to the output probabilities of the last layer neuron which corresponds to unknown pandas, and therefore, the corresponding ROC curve can be plotted (Figure 9b). At 5% false acceptance rate, the algorithm can correctly detect unknown pandas with an accuracy of 93.18%.

**Figure 10:**
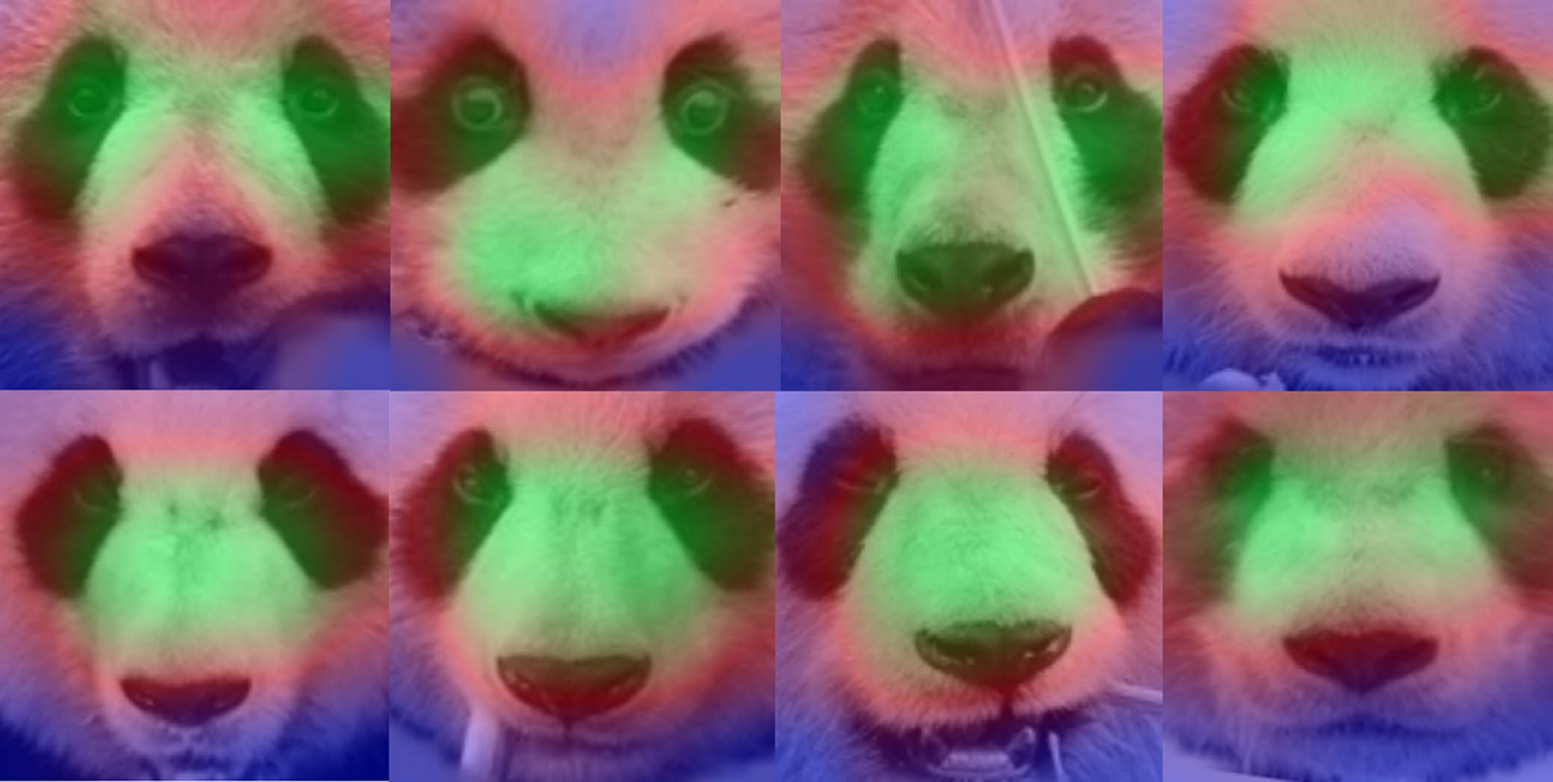
Heatmaps generated by Grad‐Cam method overlaid on panda images

What features the network learned to identify the pandas? This is a pertinent question that needed to be investigated. As in (Miao et al., 2019), the Grad‐Cam (Selvaraju et al., 2017) method is used to give insights into the network's learned features. Grad‐Cam is a widely used technique providing visual explanations of CNN based models by visualizing the gradients flowing from the final convolutional layer to the input and producing a heatmap. Grad‐Cam was used to generate heatmaps and overlay them on the input panda images. The heatmap (Figure 10) shows panda face areas that contribute the most (green) and the least (blue) in the network's prediction. These heatmaps indicate that the eyes and nose areas allow the network to distinguish between different pandas.

## 4 DISCUSSION

In the experiments, the algorithm is evaluated on challenging settings, where there are 218 pandas in the closed identification and 176 pandas in the open identification. The large numbers of pandas mean that the algorithm is more difficult to identify the pandas. However, when applying the algorithm on wild images, it is not necessary to identify these large amounts of pandas, because wild pandas are sparsely distributed in large areas and pandas are generally solitary (Guan et al., 2016). More clearly, according to the most recent panda survey (State Forestry Administration, 2015b), the density of wild pandas is 0.0684 individuals per km square and therefore, each camera installed in their habitat can only photograph several pandas. When comparing panda images taken by a particular camera, the algorithm only needs to compare them with images taken by the same camera or surrounding cameras. The number of pandas required to be matched is much lesser than the number of pandas examined in this study.

In this study, each image is considered as an independent sample and the algorithm identifies the panda in each testing image. Wild cameras, in fact, can take a video clip in each encounter. Even though the video clip can have more than 100 images for one panda, the algorithm only needs to make one decision. Thus, ensemble classification techniques, such as voting and weight sum, which can boost accuracy, can be used. In the experiments, the algorithm is examined on an extreme condition, where in each encounter, only one valid image can be used for recognition and the location of the camera is not available.

Taking frontal panda face images with good quality is an essential step to use the algorithm for panda identification. Zheng et al. showed that conspecific decoys can increase wild panda image quality (Zheng et al., 2016). Furthermore, camera traps with multiple cameras photographing the same panda from different directions can increase the chance of taking frontal panda face images. Another direction to alleviate the requirement of frontal panda face images is to extend the algorithm for handling panda face images taking from different directions.

## CONFLICT OF INTEREST

None of the authors have any conflict of interest to declare.

## AUTHOR'S CONTRIBUTIONS

P.C. provided background knowledge, including the need of panda identification, oversaw the data collection, and verified all the panda identities in each image. S.P. developed the algorithm, implemented the software, performed all the experiments, wrote the supporting document, and prepared all figures and images in the manuscript. W.M.M. developed a part of the algorithm and drafted the manuscript based on the information provided by S.P. and P.C. A.W.K.K. oversaw the algorithm development and experimental evaluation and wrote the manuscript based on the information provided by the other authors. H.S. oversaw the data annotation performed in her institute. R.H. provided background knowledge, including the need of panda identification and oversaw the data collection. Z.Z. provided background knowledge and coordinated different departments to collect the data.

## Supporting information

Appendix S1

## Data Availability

The data used in this study will be published and made publicly available at “Panda images dataset”, https://doi.org/10.21979/N9/8CYVGF, DR‐NTU (Data).

## ENDNOTE

^1^ In recent years, deep learning, deep neural networks, or convolutional neural networks gained tremendous popularity in many scientific fields. Rather than describing how they work we merely provide a reference (LeCun, Bengio, & Hinton, 2015), which is suitable for a broad spectrum of readers. If the reader is not familiar with the deep learning, it is strongly recommended to read it.
