# Supplementary material for: A study on giant panda recognition based on images of a large proportion of captive pandas
Source: Ecol Evol. 2020 Mar 10;10(7):3561–73. doi: 10.1002/ece3.6152 (PMC7141006; doi:10.1002/ece3.6152)
Supplement: Supplementary file 1 — Appendix S1 [file ECE3-10-3561-s001.docx]

Panda Recognition – Supporting Document

# **Details of the Recognition Algorithm**

A CNN model is proposed for sequential panda face detection, segmentation, landmark localization and alignment, and identity classification. The proposed network architecture is deep, and it relies on the ResNet architecture, which combats the problem of vanishing gradient by the use of skip connections. In this section, the system architecture and its components are discussed.

The proposed panda face recognition algorithm consists of 4 modules. The first one scales the input raw images taken using various cameras to either $1024\times600\times3$ or $600\times1024\times3$ depending on whether the input raw image was captured in the landscape mode or portrait mode. The first module then generates frontal panda face region proposal and crops the image to the proposed region, then passes the output to the second module. The second module scales the input to $224\times224\times3$ pixels and segments the panda face out of the image to remove the background pixels. The module is a CNN that generates a $224\times224\times1$ binary mask. When the values of this mask are multiplied with the values of the input, it segments out the background pixels and leaves the panda face in the input. The third module is a spatial transformer network which is trained to align the segmented panda faces to a reference panda face. The fourth module is a classifier that classifies the output of the third module to one of the integer class id’s assigned to the pandas.

##
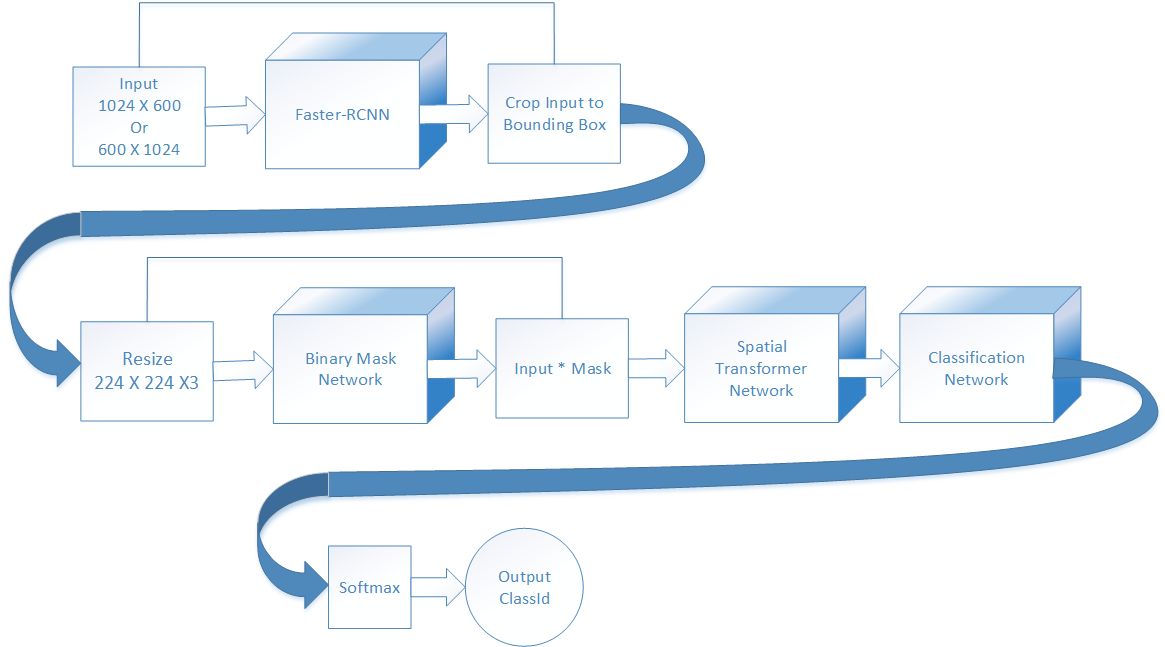


Figure 1: Network architecture of the proposed algorithm.

## **Recognition System Architecture**

The architecture initiates with a Faster R-CNN module that uses ResNet50 CNN layers. The weights of this network were trained using the COCO dataset; this pre-trained network is downloaded from the Tensorflow detection model zoo. The module does the fundamental task of detection of a frontal panda face in the input raw image. It computes the bounding box coordinates around the detected panda face. Faster R-CNN is a state-of-the-art generic object detection algorithm that consists of a region proposal network (RPN) responsible for proposing regions in the image that might contain an object, and a network to classify the objects in the proposed regions and refine the boundary coordinates of the proposed region to fit the detected object better. The module was trained to detect only the frontal panda faces in the input image.

The second module is a binary mask auto-encoder that uses several layers of a pre-trained ResNet50 model which was trained using the Imagenet dataset. A pre-trained ResNet50 model is taken and all the layers till the ResNet Layer 3 are kept, and the rest of the layers are discarded. The weights of these layers are frozen. A new convolution layer is added on the top of the modified network. Then the features are passed through an average pooling layer and a few convolution transpose layers as shown in the diagram below. The output of this network is a 2D binary mask with the shape $224\times224\times1$. When multiplying it with the input tensor, it zeros out the pixels that do not belong to the facial region.


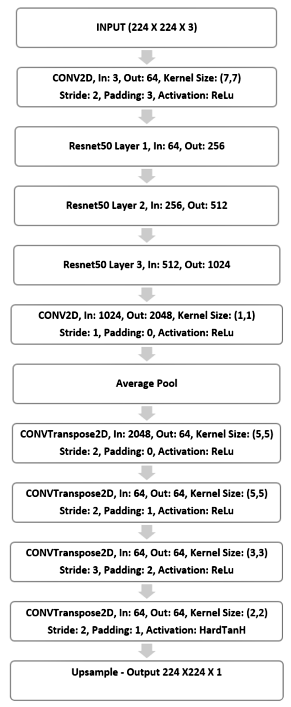


Figure 2: The binary mask network.

The third module is a spatial transformer network; the primary objective of this module is facial landmark localization and alignment. The task of this network is the regression of affine transformation parameters. A pre-trained ResNet-50 model is taken; all the layers till ResNet Layer 4 are kept, and the rest of the layers are discarded. The weight of the layers until ResNet Layer 3 is frozen. The ResNet Layer 4’s weights and all weights forward are updated during the training. Then the features are passed through an average pooling layer and followed by 2 fully connected layers. The final output layer has 6 values. The 6 values of the output layer define the parameters of the first two rows of a 3 by 3 affine transformation matrix to represent the 2D transformation of the image. The affine grid transformation is then applied to the image. The network diagram for the spatial transformer network is given below.


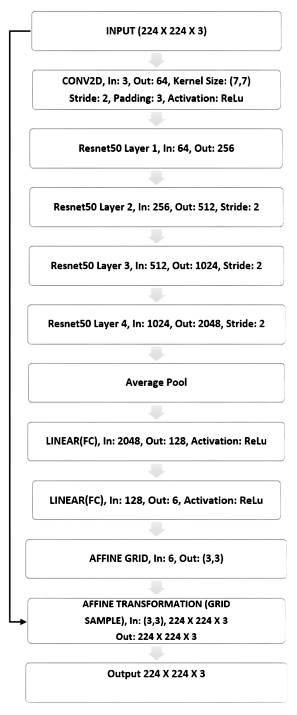


Figure 3: The spatial transformer network.

The fourth module is a standard classifier network. A standard pre-trained ResNet50 model, trained using the Imagenet dataset was downloaded, and the last layer was dropped. A new, fully connected layer was added to predict the probabilities of each panda. An integer class was assigned to each panda identity. All the weights of the classification model were unfrozen and updated during the training.

## **Training**

The networks were trained in two phases. First, the Faster R-CNN module was retrained using the raw panda images dataset. While training the Faster R-CNN network, four types of losses are calculated. The RPN regression loss is used to train whether an anchor should be chosen as a proposal; the RPN classification loss is used to train whether the anchor contains an object or not; Fast R-CNN regression loss is used to fine-tune the coordinates of the bounding boxes, and the Fast R-CNN classification loss is used to train the loss over the object class prediction. The module was trained for 200,000 epochs, and it predicted a bounding box around a single frontal panda face in the raw image which included the part/whole of a panda and surrounding objects such as trees, doors, and humans among others.

In the second phase of training, the remaining three modules were trained sequentially. The loss functions used for each module were different, calculated as per their corresponding ground truths. However, training was done in a detached manner such that the gradient of the loss calculated for the landmark localization and alignment module would not be used to update the weights of the segmentation module, and so on. The L1loss function was used for the segmentation and the spatial transformer networks. The CrossEntropyLoss function was used to calculate the loss of the classification network. The networks were trained sequentially. Manually cropped and segmented images were used for training these networks. The binary masks ground-truths were generated using the manually marked face segmentation data by binarization. Then using the manually segmented masks for the eyes and the mouth, a method was used to align the images for generating the landmark localization and alignment ground-truth dataset. Only the classification test error was used to stop the training and choose the best models; therefore if the classification error for the validation set was at the lowest at epoch 76, then all the models from epoch 76 including the segmentation model, alignment model, and the classification model were used for evaluation of the probe set as classification was the primary task. The initial learning rate of 0.01 was used for the classification network and 0.0001 for the other networks. A cosine annealing learning rate scheduler was used. SGD optimizer with a weight decay of 0.0005 for classification network. Adam optimizer was used for the mask and spatial transformer networks. All the networks were trained using Ubuntu 18.04 workstation running on an Intel Xeon(R) E5-1650 v4 CPU installed with an NVidia GTX 1080 Ti GPU and CUDA version 10.0.
